# Extracellular matrix remodeling therapeutic strategies to tackle central nervous system diseases

**DOI:** 10.4103/NRR.NRR-D-25-01562

**Published:** 2026-02-05

**Authors:** Daniel A. Domingo-Lopez, Maria Rosa Aguilar de Armas, Sergio Martin-Saldaña

**Affiliations:** 1 Instituto de Ciencia y Tecnología de Polímeros (ICTP) CSIC, Madrid, Spain; 2 Centro de Investigación Biomédica en Red de Bioingienería, Biomateriales y Biotecnología CIBER-BBN, Instituto de Salud Carlos III, Madrid, Spain

**Keywords:** central nervous system, chondroitin sulphate, chondroitinase ABC, extracellular matrix remodeling, extracellular matrix, fibronectin, heparan sulphate, hyaluronic acid, hyaluronidase, neurodegeneration, neuroinflammation, proteoglycan

## Abstract

Neurodegenerative diseases are a global burden due to the increased life expectancy. Neuroinflammation is not only a result of neurodegeneration but a key player in the initiation and onset of it. Due to the inherent complexity of these diseases, there is a need for the development of better treatments, as well as the discovery of new therapeutic targets. In this sense, knowledge about extracellular matrix remodeling after injury in the central nervous system was overseen for a century, but it has blossomed in the last three decades. Nowadays, we possess strong evidence regarding the imbalance, over-synthesis, and changes in the organization of most key components of the extracellular matrix after neuroinflammation and neurodegeneration. Thus, hyaluronic acid, chondroitin sulphate proteoglycans, or fibronectin presented an impairment in their anabolism and catabolism, which could be a cause or a consequence of the inflammatory and degenerative process. Regardless, it is clear that extracellular matrix remodeling plays a pivotal role in the onset and resolution of inflammation-driven neurological disorders by creating a non-permissive niche for self-restoration of the neural homeostasis. Despite being an emerging area of study, extracellular matrix changes have been explored in the last decades in the central nervous system to shed light on their potential as diagnostic markers as well as therapeutic targets. The extracellular matrix fingerprint in diseases such as multiple sclerosis, Alzheimer’s disease, Parkinson’s disease, or stroke has been described both in preclinical models and in post-mortem clinical samples. Herein, we provided an overview of the state of the art of extracellular matrix components and function in the central nervous system, both under homeostasis and in neurodegeneration. Then, we critically revise the therapeutic efforts targeting the extracellular matrix, aiming to tackle neurodegenerative disorders. Altogether, this review contextualizes the current understanding of extracellular matrix in the central nervous system and its remodeling after neuroinflammation, its role in disease onset and resolution, and how this knowledge is being applied to the development of new therapeutic approaches.

## Introduction

Neuroinflammation plays a pivotal role in neurodegenerative diseases, not only as a consequence of it but as one of the main aspects in the disease onset and progression. Neurological conditions are nowadays the main cause of illness worldwide according to the World Health Organization, mostly due to the increase in life-expectancy (Steinmetz et al., 2024). Furthermore, 80% of deaths and health loss related to neurodegeneration occur in low- and middle-income countries (Steinmetz et al., 2024). Due to the high complexity of the central nervous system (CNS) during homeostasis and in the course of neurodegenerative disorders, there is a clinical need for new therapies that could resolve this world burden.

The study of the pathophysiology of extracellular matrix (ECM) in neuroinflammatory-driven diseases and disorders has blossomed in the last three decades. In the case of the CNS, the ECM is highly specialized, presenting a unique composition that consists mostly of nonfibrillar components. ECM is being secreted by neuroepithelial cells in early developmental stages, when it represents 40% of the total brain volume, and by glial cells and neurons during CNS maturation, accounting for 10%–20% of the total volume (Benarroch, 2015). Furthermore, its composition is dynamic during development of the CNS, being temporarily and spatially regulated to aid neurogenesis, axonal growth, cell migration, and differentiation (Benarroch, 2015). The ECM in postnatal stages regulates synaptic plasticity and is remodeled both under homeostasis and pathological conditions, playing a key role in CNS biology during the lifetime. The body of research proving ECM remodeling in Alzheimer’s disease (AD), Parkinson’s disease (PD), stroke, traumatic brain injury (TBI), spinal cord injury (SCI), or multiple sclerosis (MS) suggests potential as a diagnostic marker and as a therapeutic target. Herein, we discuss the current knowledge of ECM components and function in the CNS, both under homeostasis and in a pathological context. Then, we critically review the current therapeutic efforts targeting ECM directly and in an indirect way, aiming to tackle neurodegenerative disorders. Altogether, this review contextualizes the current understanding of ECM in the CNS and its remodeling after neuroinflammation, its role in disease onset and resolution, and how this knowledge is being used for the development of new therapeutic approaches.

## Literature Retrieval Strategy

Literature searches were performed using PubMed and Web of Science specially focused, but not limited, to publications from 2000 to 2025. The following keywords using various combinations were used to ensure broad coverage of the topic: “central nervous system” AND “extracellular matrix” AND “remodeling/remodelling” AND (“multiple sclerosis” OR “Alzheimer’s disease” OR “Parkinson’s disease” OR “Huntington’s disease” OR “stroke” OR “spinal cord” OR “chondroitinase ABC” OR “hyaluronidase”). Articles were included on the basis of their relevance to the topic, with a focus on therapeutic approaches aiming to remodel the ECM in CNS disorders. Further screening of reference lists was conducted to identify additional relevant studies.

## Extracellular Matrix Components in the Central Nervous System

ECM is a key component of every human cell and tissue environment, playing a structural and functional role, being the primary regulator of cellular processes such as cell adhesion, cell differentiation and proliferation, metabolism, programmed cell death, and homeostasis maintenance (Goswami et al., 2021; Martin-Saldaña et al., 2022). From a structural point of view, ECM provides neural cells with anchorage points, facilitating cell organization within distinct CNS regions. Chemically, ECM provides various molecular signals that guide cell growth, activity, and survival (Lau et al., 2013). ECM is secreted by the tissue-resident cells and actively communicates with cells through surface receptors and/or matrix effectors. Moreover, ECM turnover is a dynamic process that occurs on a daily basis (Tobisawa et al., 2021), evolving its structural composition during the lifespan and in response to pathology (Lau et al., 2013).

**Figure 1:**
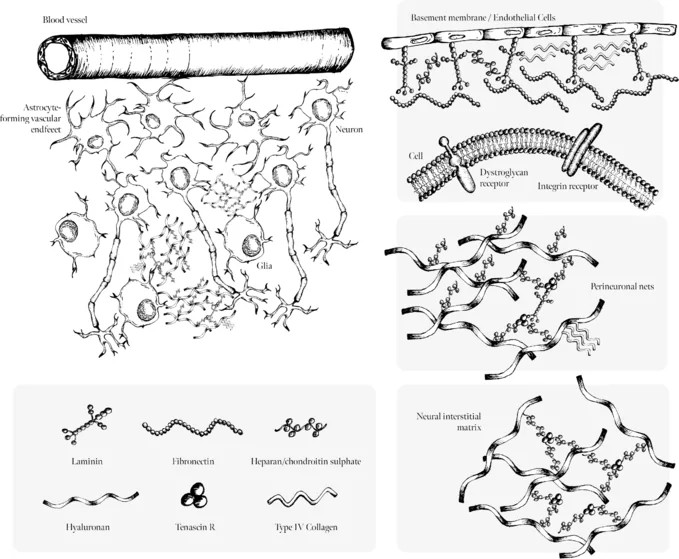
Main compartments of the extracellular matrix of the central nervous system. Components of the extracellular matrix in the central nervous system are arranged into three main compartments, namely the basement membrane that surrounds cerebral vessels, the perineuronal nets that are condensed around neural bodies and their dendrites, and the diffusely distributed interstitial matrix, which is constituted by neurons and glial cells, the parenchyma of the central nervous system.

The ECM in the CNS differs from ECM of other tissues in terms of composition and organization (**Table 1**). Unlike other tissues where ECM is generally composed by fibrillary proteins such as collagens, ECM in the CNS is mostly composed of proteoglycans (PGs) and glycosaminoglycans (GAGs). Interestingly, collagens are not abundant in the CNS, being present only on the basal lamina or basement membrane (BM). ECM in the CNS presents three different organizations/compartments with particular structural and functional implications: the BM, the interstitial ECM of the CNS parenchyma, and the perineuronal nets (PNNs) (**Figure 1**).

**Table 1:** Main components of ECM in the CNS, functions, and main localization

ECM component	Description	Main function	Location
Aggrecan	CSPG type of the lectican family	Contributes to structural integrity and provides cues for neural growth and synaptic plasticity	PNN
Agrin	HSPG type, which is widely expressed in various spliced forms	Key to support functions of synapse/astrocytes	Widely distributed
Brevican	CNS-specific type of CSPG, mainly expressed by astrocytes, pericytes, and neurons	Key role for the structure of the PNN	PNN
Chondroitin sulphate	GAG polymer formed by repeated units of N-acetylgalactosamine and glucuronic acid disaccharide	Forms CSPGs	Widely distributed
Collagen	Fibrillar protein providing a scaffold for cells	Crucial structural component of the BM. Provides organization and stability, and regulates neural stem cells differentiation	Mainly in BM, but it is present in the interstitial ECM
Elastin	Fibrillary protein	Structural component of the BM providing elasticity to the vessels of the BBB	Mainly in BM, also present in the interstitial ECM
Fibronectin	Glycoprotein dimer composed of two monomers linked by a disulfide bond. It is secreted by ECs, astrocytes, and pericytes	Substrate for cell adhesion. Attachment and migration of neural cells. Guidance of axon growing during development	Widely distributed
Heparan sulphate	GAG polymer formed of repeated units of the N-acetylglucosamine and glucuronic Acid disaccharide	Forms HSPGs	Widely distributed
Hyaluronic acid	GAG polymer formed of repeated units of nonsulphated nacetylglucosamine and glucuronic acid disaccharide	Key component of PNNs and interstitial ECM considered as the ECM backbone due to the linkage of CSPG and HSPG to its chain. Contributes to ECM’ hydration and structural integrity. Modulates cell migration and cell behavior. Its biological activity is mainly mediated via CD44 receptors	PNN and interstitial ECM
Laminin	Family of glycoproteins	Confers structural integrity, mostly on the BM. Crucial for cell adhesion, tissue organization and neural development. Responsible of synaptic functions and plasticity	BM and interstitial ECM
Matrix metalloproteases	Zinc-dependent proteases	Key role in degrading and remodeling ECM. Neurogenesis, neural plasticity and development. Their expression and activity are altered under pathological context	Widely distributed
Neurocan	Specific type of CSPG of the lectican family in the CNS; formed by neurocan core-protein and chondroitin sulphate	Binds to HA and tenascin-R/C. Maintains neurites growth and synaptic function	Widely distributed
Nidogen	Sulphated glycoprotein	Structural integrity of the ECM by binding collagen and laminin. Plays role in axon guidance	Mainly in BM
Perlecan	HSPG	Interacts with collagen and laminin for the structural integrity and stability of BM	BM
Tenascin	ECM glycoprotein’ family	Interacts with Fn, brevican, neurocan, HA, and integrins. Crucial for structural support and tissue formation. Responsible of synaptic function	PNN and interstitial ECM
Versican	CSPG	Binds to HA and Fn. Responsible for maintaining neurites growth and neuronal migration	Interstitial ECM

Data are sourced from Li et al. (2021), Anwar et al. (2022), and Nazir et al. (2025). BBB: Blood–brain barrier; BM: basement membrane; CNS: central nervous system; CSPG: chondroitin sulphate proteoglycan; EC: endothelial cell; ECM: extracellular matrix; Fn: fibronectin; GAG: glycosaminoglycan; HA: hyaluronic acid; HSPG: heparan sulphate proteoglycan; MMP: matrix metalloproteases; PNN: perineuronal network.

The BM acts as a protective barrier between the brain parenchyma and the endothelial cells that form the blood vessels. The main role of BM in the CNS consists of maintaining the blood–brain barrier (BBB). Thus, BM forms a sheet-like structure underneath the endothelial cells covering the pial intracranial vessels and glial cells, separated by pericytes, both in the brain and spinal cord. BM is composed of collagen (Collagen-IV), laminin, nidogen, heparan sulphate proteoglycans (HSPGs), and fibronectin (Fn) (Xu et al., 2019). Thus, BM represents an ECM structure lining the parenchymal side of CNS microvessels, connecting the endothelial cell compartment to astrocytic endfeet.

The PNN is a condensed mesh-like layer of ECM that surrounds the soma and dendrites of neural cells, acting as a platform to make synaptic connections stable (Fawcett et al., 2019). Most PNNs can bind the lectin Wisteria floribunda agglutinin, which has been used as a PNN marker in histological analyses (Ge et al., 2024). Main components of PNNs are members of the lectican family of chondroitin sulphate proteoglycans (CSPGs) such as aggrecan, brevican, neurocan, and versican. Furthermore, a backbone hyaluronic acid (HA) to which lecticans bind via their link domain is prevalent in the composition of PNNs, as well as glycoprotein tenascin-R that links lecticans to each other (Fawcett et al., 2019). It is also thought that the HA–CSPGs– tenascin complex, deposited on the neuronal cell surface, forms a protective barrier surrounding axons and dendrites (Evans et al., 2024).

The interstitial matrix is made of a loose mesh of HA, CSPGs, tenascins, and proteins (**Figure 1**). Furthermore, the interstitial matrix comprises small amounts of fibrous proteins such as collagens and elastin, as well as adhesive glycoproteins, such as laminins and Fn. Altogether, the interstitial matrix provides structural and chemical support, connecting neurons and glial cells with the vasculature (Nazir et al., 2025).

## Extracellular Matrix Remodeling under Central Nervous System Pathology

Acute and chronic insults trigger alterations to the ECM of the CNS, being its extent dependent on the severity and chronicity of the injury. Furthermore, the inherent complexity of the CNS comes with a plethora of different pathophysiology depending on the disorders, which affects the ECM. ECM molecules can be both the cause and the consequence in CNS pathogenesis, and their remodeling, or lack of it, determines the outcome and progression of the disease. It is well established that glial cells play a pivotal role in ECM remodeling under homeostasis as well as during CNS pathology. Astrocytes and microglia are the primary glial cells in the brain that regulate neuroinflammation through a bidirectional “crosstalk.” Astrocytes are the main players in the synthesis of ECM components such as HA or CSPGs. Studies about microglia-ECM crosstalk and its impact on processes such as synapse remodeling or injury repair have blossomed in the last five years (Strackeljan et al., 2021; Cangalaya et al., 2024; Wareham and Calkins, 2025). Emerging evidence highlights the crucial role of ECM in microglial behavior both in physiological and pathological contexts, which has been recently revised by Wareham and Calkins (2025). Indeed, microglia are highly sensitive to minor perturbations during homeostasis, requiring highly complex interactions with the ECM (Cangalaya et al., 2024). This ECM remodeling is in part mediated by microglial phagocytosis (Wareham and Calkins, 2025), while the integrity of the ECM seems to play a key role in the ability of microglia to remodel synapses (Cangalaya et al., 2024). However, the finely tuned ECM downregulation is disrupted in CNS disease, partially due to microglial activity. ECM alterations have a direct effect on microglial function. Thus, ECM-cell interaction has been recently described as an emergent critical regulator of brain homeostasis and disease progression (Wareham and Calkins, 2025).

The CNS is protected by a tight vascular barrier due to the highest sensitivity of neurons to relatively harmless agents. The BBB, which consists of a perfectly orchestrated conjunction of cells (endothelial cells, pericytes, astrocytic endfeet, neuronal processes, and microglia) and the BM, can be altered during neurodegeneration (Romero-Ben et al., 2025). As a matter of fact, its leakage is commonly used as a diagnostic sign for neurodegenerative diseases, such as PD (Al-Bachari et al., 2020) or AD (Farrall and Wardlaw, 2009). Conversely, understanding if changes in BM components are a cause or consequence of neurodegeneration remains an open question. Furthermore, the BBB integrity seems to be compromised in Huntington’s disease, an autosomal disease caused by a mutation in the gene for the Huntingtin protein, by impairing the ability of astrocytes and endothelial cells to express integrin. Therefore, the ECM-integrin interface is affected, contributing to molecular and cellular pathogenesis of Huntington’s disease (Hernandez et al., 2023).

The most evident cases of ECM changes after injury of the CNS are those caused by traumatic or ischemic events. The ECM suffers dramatic and, in most cases, permanent changes after SCI (Alizadeh et al., 2019), stroke, and TBI (Muneer et al., 2016). Neurons fail to re-form the network after the injury due to the loss of ECM integrity and structure in the acute phase, and because of the ECM scarring in the chronic phase (Chmelova et al., 2023). Thus, a non-permissive ECM with abnormal accumulation of some of the components, such as HA and CSPGs, is formed, which limits the ability of neurons and glial cells to self-restore tissue homeostasis. Furthermore, the breakdown and detachment of BM in cerebral ischemia are well reported, mostly mediated by the overexpression of matrix metalloproteinases (MMPs), particularly MMP2 and MMP9 (Yang et al., 2013; Wang et al., 2024).

The research assessing alterations in the ECM in terms of expression and composition has blossomed in the last two decades, and it has been extensively reviewed (Pintér and Alpár, 2022). In this section, we provide a succinct overview of the main changes in ECM in the most prevalent CNS diseases. Therefore, the abnormal expression of components in the ECM, such as HA, CSPG, Fn, or MMPs, has been studied in depth in some of the most permanent CNS diseases, such as AD, MS, PD, epilepsy, and schizophrenia, suggesting a crucial role in their pathogenic profile.

## Abnormal Production of Hyaluronic Acid in Neurodegeneration

One key change in the ECM during neurodegenerative diseases is the increase in HA synthesis. Particularly, low molecular weight HA is accumulated in demyelinated lesions during acute lesions, while high molecular weight (HMW) HA is accumulated in the demyelinated axons during chronic lesions (Back et al., 2005). Thus, during chronic CNS lesions, HA accumulations forms a scar inhibiting oligodendrocyte precursor cell maturation, leading to impaired remyelinization (Back et al., 2005). As a consequence, increased levels of HA in the cerebrospinal fluid of patients have been demonstrated to be a useful diagnostic marker in MS (Nielsen et al., 2012; Zhang et al., 2021). Additionally, HA levels in urine and skin are increased in patients with sporadic amyotrophic lateral sclerosis compared with healthy subjects (Ono et al., 1996; Apolloni and D’Ambrosi, 2022).

Furthermore, HA has been reported to accumulate mainly at the perimeter of amyloid-beta (Aβ) plaques and hyper phosphorylated tau tangles in AD (Reed et al., 2019). Moreover, hyaluronan synthase 2 (HAS2), the most abundant HA synthase in the CNS, is significantly overexpressed in AD, which causes an impaired oversynthesis of the GAG (Reed et al., 2019). Similarly to what occurs in MS, low molecular weight HA is synthesized, potentially triggering inflammation, while HMW HA accumulation in Aβ plaques is associated with advanced stages of pathology. Particularly, HA-tumor necrosis factor-stimulated gene-6 interaction appears to be increased in AD, potentially aiming to moderate the inflammatory response.

A recent study about the potential role of HA in PD has been reported in an *in vitro* model of transgenic SH-SY5Y human neuroblastoma cells expressing the PD-causing familial mutation in VPS35 (D620N), which inhibits autophagy (Rahman et al., 2020). Researchers reported that hyaluronan-mediated motility receptor or CD44 knockouts resulted in upregulated autophagy in cells expressing VPS35 wild-type. Interestingly, hyaluronan-mediated motility receptor (–/–) resulted in rescue of autophagy dysfunction by VPS35 D620N, which suggests a potential pathogenic role of the receptor and HA signaling in PD (Rahman et al., 2020). However, preclinical and clinical assessments are still needed to understand the role of HA in this CNS disease.

## Proteoglycan Overexpression in Neurodegenerative Diseases

Similarly to what happens with HA, a big body of research evidences PGs accumulation during neurodegeneration. This phenomenon might be related to an attempt to maintain CNS function or, conversely, to trigger tissue breakdown. Since this question remains unsolved, future research must aim to shed some light on our knowledge related to the functional role of these spatiotemporal PG changes between homeostasis and pathology.

In MS, lecticans such as aggrecan, versican, and neurocan, as well as dermatan sulphate-proteoglycans are accumulated in acute lesions alongside astrogliosis (Sobel and Ahmed, 2001). This pathological phenomenon generates a non-permissive environment for axonal growth and remyelination (Sobel and Ahmed, 2001). Furthermore, CSPG and dermatan sulphate-proteoglycan overexpression results in macrophage and astrocyte activation, enhancing the inflammatory response (Hibbits et al., 2012; Yu et al., 2022). The resulting astrogliosis and macrophage activation trigger CSPGS depletion, further contributing to neuroinflammation.

Neurite degeneration, cell adhesion, and growth factor distribution are negatively impacted by the accumulation of CSPG in AD, similarly to what is observed in SCI (Monnier et al., 2003). The presence of CSPGs in neurofibrillary tangles and plaques has been reported both in preclinical models and humans (Dewitt et al., 1993). Interestingly, despite the inhibiting environment created by CSPGs accumulation, which restricts plasticity, the ECM remodeling seems to protect neurons from degeneration.

Other PGs, such as the HSPGs agrin, accumulate as well in AD patients, which enhances Aβ fibril formation (Snow et al., 1988). Furthermore, HSPGs accumulation on Aβ plaques was proven to be co-localized with hyperphosphorylated tau, regulating cellular immune responses to tau protein monomers (Song et al., 2022). Interestingly, HSPG accumulation occurs at early stages of plaque development, suggesting a key role in its onset (Snow et al., 1988). Moreover, in PD, agrin seems to colocalize with α-synuclein in Lewy bodies both in animal models (Chen et al., 2022) and in human tissues (Raghunathan et al., 2020). Recently, the accumulation of HSPGs and HA in the serum of neuromyelitis optica patients showed big potential as a diagnostic biomarker and a prognostic one (Zhang et al., 2021).

## Role of Glycoproteins in Central Nervous System Pathology

Glycoproteins and their distribution changes are crucial in many neurodegenerative disorders, such as MS, AD, or PD.

Tenascin-C is involved in the neuroinflammatory processes of AD. When the brains of 14 patients were analyzed post-mortem, this glycoprotein was largely deposited in the cortical grey matter, overlapping and surrounding cored Aβ plaques. These tenascin-C deposits were associated with reactive glial cells and phosphorylated tau-containing neurites. Interestingly, the diffuse plaques that did not present astrocytes and reactivity of microglia did not show tenascin-C accumulation, further suggesting its potential as a diagnostic marker and a therapeutic target (Mi et al., 2016).

Tenascin concentrations decrease in active lesions of MS, but seem to be unaltered in chronic ones (Gutowski et al., 1999). Conversely, Fn and vitronectin levels are increased (Stoffels et al., 2013). Tenascins, both C and R, promote demyelination through the oligodendrocyte precursor cells, as well as promote inflammation by activating microglia (Bauch and Faissner, 2022). Fn leaks from the BM and is also over-synthesized by CNS cells, mostly mediated by neuroinflammatory processes, subsequently inhibiting remyelination (Stoffels et al., 2013).

Additionally, laminin levels are altered in MS (Wu et al., 2009). AD is accumulated in amyloid plaques (Rodin et al., 2020) and PD. Particularly, in PD, recent results showed the potential of laminin for promoting dopaminergic neuron survival (Zhang et al., 2017).

Altogether, it is clear that ECM suffers a remodeling in neurodegeneration, mostly associated with inflammation. Moreover, ECM modulating enzymes such as MMPs and tissue inhibitors of MMPs are differentially expressed in homeostasis and disease, playing key roles in ECM remodeling (Kazemi et al., 2025). Despite the increasing knowledge on the fingerprint of ECM components in homeostasis and pathology, we still need to understand the potential of these changes in diagnosis and therapy. In the next section, we critically discussed the most recent therapeutic approaches targeting ECM remodeling in CNS diseases and their limitations.

## Therapeutic Approaches

As previously discussed, pathogenic ECM changes, such as accumulation of CSPGs, HA, Fn, and altered ECM crosslinking/morphology, can contribute to glial scarring, synaptic dysfunction, and neuroinflammation (Alizadeh et al., 2019).

**Figure 2:**
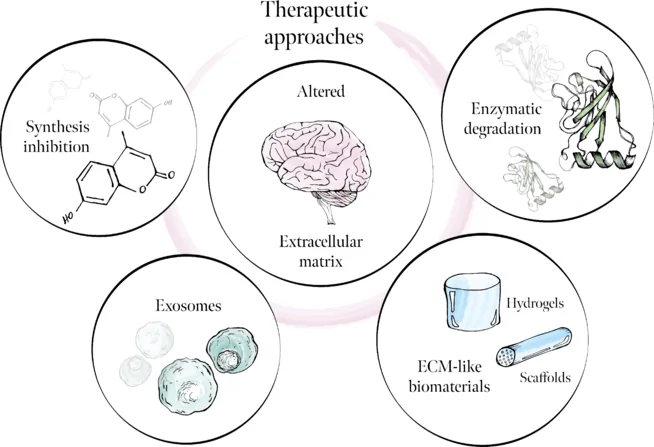
Therapeutic approaches aiming to reprogram extracellular matrix (ECM) remodeling. The available toolbox aiming to tackle central nervous system disorders through ECM remodeling is focused on enzymatically degrading the non-permissive ECM, regulating ECM components synthesis, or promoting a regenerative phenotype to restore neural plasticity by using biomaterials and exosomes.

Traditionally, therapies for these conditions have focused on inflammation suppression and/or neuroprotection. However, more recent strategies aim to restore ECM homeostasis using advanced technologies that include (1) biomaterials (e.g., hydrogels, scaffolds, and peptides) or (2) small-molecule modulators (e.g., enzymatic inhibitors, therapeutics, gene therapy, and exosomes). These approaches aim to reprogram the local injury microenvironment, degrade ECM inhibitory components, regulate synthesis or receptor-mediated signaling, reduce pro-inflammatory cytokines, and promote a regenerative phenotype to restore neural plasticity (**Figure 2**). Here, we discuss several preclinical and translational studies across the main neurological conditions in which biomaterial-based approaches and small-molecule modulators are used to target ECM remodeling, reduce neuroinflammation, and promote functional recovery.

## Spinal Cord Injury and Traumatic Brain Injury

SCI and TBI are devastating lesions caused by CNS traumas that induce profound changes in the ECM. In both SCI and TBI, the initial mechanical insult unleashes a secondary inflammatory cascade, triggering microglia activation, macrophage infiltration, and release of proinflammatory cytokines (Griffiths et al., 2020). This can lead to the upregulation of matrix-degrading enzymes such as MMPs. These enzymes degrade key ECM components such as HA, CSPGs, laminin, or collagen, resulting in BM disruption, BBB or bloodspinal cord barrier breakdown, and deposition of inhibitory scar matrix. In SCI, this contributes to glial and fibrotic scar formation that physically and chemically blocks axonal regrowth, while in TBI, ECM degradation alters signaling, inflammatory regulation, and circuit remodeling, ultimately impeding neural repair and functional recovery (George and Geller, 2018; Kjell and Götz, 2020).

In SCI models, the use of HA hydrogels incorporating decellularized ECM derived from astrocytes has demonstrated powerful effects and therapeutic potential on the injury microenvironment. In these studies, the ECM was harvested from mouse embryonic stem cell-derived protoplasmic (gray matter–like) astrocytes or fibrous ECM (white-matter) astrocytes and embedded within HA hydrogels, with and without coimplantation of V2a interneurons. Two weeks postimplantation, the ECM hydrogels containing HA and protoplasmic/fibrous astrocyte ECM significantly reduced glial scar formation (as assessed by glial fibrillary acidic protein [GFAP] staining), decreased CSPG levels (CS56 labelling), and lowered macrophage/microglia infiltration (CD11b^+^, ED1^+^ cells) within and around the lesion (500 µm surrounding the lesion). Interestingly, the reduction in scar size, enhancement of axonal penetration into the lesion, and attenuation of immune cell staining were specific to protoplasmic ECM (P-ECM:HA, grey matter), while fibrous ECM hydrogels (F-ECM:HA, white matter) failed to confer these benefits, highlighting the importance of region-specific ECM composition in the therapeutic outcomes. For instance, P-ECM:HA induced motoneuron growth *in vitro*, with longer neurites (20.465 ± 2415 pixels/nucleus) compared with F-ECM:HA (10.276 ± 1423 pixels/neurons); and *in vivo*, with longer axonal growth into SC lesions, assessed by β-tubulin III area (14% ± 1.0% in P-ECM:HA *versus* 8.0% ± 0.6% in F-ECM:HA *versus* 9.2% ± 1.9% in sham). Furthermore, scaffolds supporting V2a interneuron transplantation promoted neurite outgrowth both within the hydrogel and adjacent host tissue. This study highlights the therapeutic potential of combining HA scaffolding with bioactive, region-specific astrocyte ECM to directly modulate the inhibitory changes in the post-injury ECM environment and promote a regenerative tissue microenvironment (Thompson et al., 2018).

Hydrogels can be combined with small (bio)molecules to enhance therapeutic outcomes. For instance, Wang et al. (2025) recently developed a photocrosslinkable HA methacryloyl hydrogel capable of releasing interleukin-10 (IL-10) for treating SCI in rat models. Importantly, beyond immunomodulation, the bioactive scaffold influenced the injured ECM by reducing astrocyte activation (glial fibrillary acidic protein; GFAP^+^ area) and decreasing deposition of inhibitory proteoglycans such as CSPGs within the lesion. The IL-10-loaded hydrogel also supported preservation of ECM structure (including laminin and Fn around the lesion boundaries), maintaining a more permissive microenvironment for axon growth. These ECM-level changes coincided with macrophage polarization toward M2 (CD206^+^), reduced pro-inflammatory cytokines, and improved motor and sensory recovery, indicating that the HA methacryloyl-IL-10 platform could recalibrate the immune milieu and preserve ECM integrity to promote repair (Wang et al., 2025).

Another interesting therapeutic element that has been recently investigated for SCI and TBI is extracellular vesicles (EVs), due to their robust potential to modulate ECM and inflammation. However, the clinical use of EVs is hindered by some limitations, including short half-life, poor accumulation, rapid clearance, and difficulty in targeting specific tissues. In this context, EVs can be combined with ECM-based hydrogels to overcome these problems and enhance their efficacy by maintaining their bioactivity, protecting them from rapid clearance, and facilitating their specific release at the target site. Additionally, the combination of ECM-based hydrogels and EVs could provide a synergistic effect, acting as a drug delivery platform while enhancing ECM modulation by serving as a material source for remodeling. This platform has been reviewed by Nazerian et al. (2023) in the spinal cord regeneration, exploring different strategies that combine various materials (HA, gelatine, PLGAs, collagen, and fibrin) and EVs sources (MSCs, NSCs, USCs, M2 microglia, and M2 macrophages). A relevant example of the combination of hydrogels and EVs was depicted by Deng et al. (2025), describing HA hydrogels encapsulating MSC-EVs, which, upon implantation, reduced collagen and CSPG deposition, creating a suitable microenvironment for axonal regeneration. Additionally, these systems promoted microglial/macrophage polarization toward the anti-inflammatory M2 phenotype (shown as an increase in M2 macrophage markers Arg1, Cd206, and IL-10 and a decrease in M1 macrophage markers Nos2, Ccr7, and Cd86), fostering axonal growth and functional recovery in SCI models (Deng et al., 2025).

This therapeutic platform is not limited to SCI; it has also been explored for other neurological disorders, including TBI. For instance, Liu et al. (2023) developed a HA–collagen hydrogel integrated with bone marrow mesenchymal stem cell-derived exosomes to enhance repair following TBI. This biomimetic system not only mimics the native brain ECM but also provides sustained local release of EVs, promoting angiogenesis and neurogenesis. The HA–collagen hydrogel integrated with bone marrow mesenchymal stem cell-derived exosome scaffold significantly reduced reactive astrogliosis and glial scarring, indicating active modulation of the inhibitory ECM environment. Moreover, it facilitated the recruitment and differentiation of endogenous neural stem cells into neurons and oligodendrocytes, supporting synapse formation and improving axonal regeneration. These effects collectively contributed to enhanced brain structural remodeling and functional recovery in a rat TBI model. These analyses demonstrate the potential of combining ECM-mimetic hydrogels with exosome-based therapies to actively remodel the post-injury ECM, promoting a regenerative microenvironment conducive to neural repair (Liu et al., 2023).

One of the major approaches in the remodeling of ECM for the treatment of neurological conditions and neuroinflammation is based on the use of enzymes that can degrade ECM components. Particularly, the therapeutic use chondroitinase ABC (ChABC) has been widely explored at different levels, ranging from preclinical to clinical studies. ChABC is a bacterial enzyme that cleaves the glycosaminoglycan side chains of CSPGs, which in glial scars act as ECM inhibitors. A 2018 systematic review and meta-analysis by Koh et al. (2018) evaluated preclinical ChABC interventions in acute brain injury models and reported an average 49% improvement in neurological recovery across studies, suggesting that CSPG digestion reduces astroglial scarring, promotes neurite sprouting, and selectively strengthens sprouting neural circuits. Complementing this, Cunha et al. (2025) highlighted delivery innovations in SCI models, including hydrogels, nanoparticle carriers, stem cell-based enzyme production, and viral vectors, aiming to stabilize ChABC at body temperature and increase its availability in lesions. These delivery platforms improve CSPG degradation, reduce inhibitory ECM density, and enhance synergistic outcomes on their own or when combined with growth factors or neural cell transplantation. Several therapeutic strategies involving ChABC have been investigated for the different neurological pathologies. A summary of some of them can be found in **Table 2**, describing the therapy and delivery route, as well as the main findings.

**Table 2:** Therapeutic approaches involving the use of chondroitinase ABC (ChABC) for different neurological disorders

Therapy/Delivery method	Condition	Main findings	Reference
ChABC + AAV-NT3/intraspinal microinjection immediately after injury	SCI (contusion)	ChABC-mediated CSPG digestion with AAV-NT3 delivery strengthened synaptic connections, increased serotonergic fiber innervation around motoneurons, and improved locomotor performance, linking ECM degradation to enhance plasticity	Hunanyan et al., 2013
ChABC/intrathecal or via thermostabilized hydrogel	SCI	CSPG digestion in glial scar facilitated axon sprouting, regeneration of both ascending/descending tracts, improved BBB integrity when combined with rehabilitation	Suzuki et al., 2017
Lentiviral ChABC (LV-ChABC)/local intraspinal gene delivery	SCI	Enzymatic CSPG digestion enabled microglia/macrophage phenotypic conversion from pro-inflammatory to pro-repair via TLR4 signaling, accelerating immune resolution and supporting ECM remodeling	Francos-Quijorna et al., 2022
ChABC/microinjection adjacent to cuneate nucleus	Dorsal column lesion (in primates)	CSPG digestion of ECM enabled reactivation of somatosensory cortical representation and regeneration of afferent signaling	Bowes et al., 2012
ChABC/local microinjection into spinal cord (cervical region)	Stroke (delayed post stroke, aged rats)	CSPG removal from PNN and glial scar, promoted sensorimotor recovery and corticospinal tract sprouting even in aged animals	Soleman et al., 2012
ChABC vs. glypican vs. neurocan/direct infusion into infarct cavity	Stroke (infarct cavity)	ChABC removes inhibitory CSPGs; glypican adds permissive ECM cues (HSPG) that upregulate FGF2 and support neurite outgrowth; neurocan alone did not show any recovery	Hill et al., 2012
Mutant ChABC (ChASE37) encapsulated in affinity-released hydrogel/epicortical injection	Stroke (rat brain)	A redesigned, thermostable enzyme degrades CSPGs in vitro and in vivo, enhancing hiPSC-derived neuron adhesion, axon sprouting, and sustained CSPG clearance around the lesion	Khait et al., 2025
Immobilized chondroitinase ABC on porous silicon nanoparticles (ChABC@PSi)/intracerebral injection	MS-like demyelination (cuprizone model)	CSPG degradation reduced astrogliosis, decreased demyelinated area, increased oligodendrocyte lineage cells and enhanced remyelination; demonstrating that ECM remodeling supports repair in MS models	Rezaei et al., 2020
ChABC/direct cortical injection (perirhinal cortex)	Alzheimer's disease (tauopathy model)	PNN digestion (CSPG removal) using ChABC reactivated synaptic plasticity and restored memory function despite persistent tau pathology	Yang et al., 2015

AlAAV-NT3: Adeno-associated viral (AAV) vector-mediated prolonged delivery of neurotrophin NT-3; ChABC: chondroitinase ABC; CSPG: chondroitin sulphate proteoglycan; ECM: extracellular matrix; FGF2: fibroblast growth factor-2; hiPSC: human induced pluripotent stem cell; HSPG: heparan sulphate proteoglycan; MS: multiple sclerosis; PNN: perineuronal network; SCI: spinal cord injury; TLR4: Toll-like receptor 4.

Several pivotal studies have tried to demonstrate the mechanism behind ChABC activity and its ability to remodel the inhibitory lesion environment. Barritt et al. (2006) showed that intrathecal application of ChABC in rodent SCI models enables robust sprouting of injured corticospinal and intact serotonergic axons into regions previously rich in CSPGs, without inducing neuropathic pain or allodynia (neuropathic pain caused by non-painful stimuli. Furthermore, Francos-Quijorna et al. (2022) revealed that CSPG accumulation prevents the resolution of inflammation by engaging Toll-like receptor 4 on immune cells. Therefore, ChABC-mediated removal of CSPGs not only permits axonal regrowth but also facilitates conversion of macrophages/microglia toward a pro-repair M2 phenotype and reduces inflammatory cytokine expression (IL-1β and tumor necrosis factor-α), reinforcing ECM modulation as a key driver of both structural and immunological recovery (Francos-Quijorna et al., 2022).

Another interesting enzymatic modulation approach consists of the use of hyaluronidase, a natural enzyme that controls HA homeostasis by degrading it. Considering that HA could be overexpressed and accumulated in neurological disorders, enhancing its “natural” enzymatic degradation could help restore healthy levels and recover normal tissue functioning. Hyaluronidase is particularly valuable in neuroinflammation, especially aiming to target the role of HA in tissue edema and inflammatory signaling. After TBI, damaged tissue elevates levels of both HAS and hyaluronidase enzymes, generating low-molecular-weight HA fragments that act as damage-associated molecular patterns. These fragments bind to Toll-like receptor 2/4 receptors on microglia and macrophages, activating nuclear factor kappa-beta and inflammasome pathways, thereby amplifying cytokine release and secondary injury (Xing et al., 2014). Washington et al. (2020) demonstrated in a mouse-controlled cortical impact model that administration of intracerebroventricular hyaluronidase (1 U/mL) significantly reduced hippocampal edema, dropping T₂ magnetic resonance imaging signal intensity from approximately 29% to approximately 14% and reducing water content in wet/dry weight analysis without compromising BBB integrity or motor and cognitive function. These findings were correlated with a significant decrease in the GAG content (determined by dimethylmethylene blue assay) in cortical explants, when comparing treated mice (5.46 ± 1.15 µg GAG/mg dry weight) with controls (7.76 ± 1.87 µg GAG/mg dry weight). Hyaluronidase works by enzymatically degrading HA, effectively reducing the negative charge load in the ECM (fixed charge density), which alleviates the osmotic pressure. By removing HA and preventing the formation of pro-inflammatory fragments, hyaluronidase not only alleviates damage caused by fixed charge density-mediated swelling, but also interrupts a key driver of neuroinflammatory signaling.

## Stroke

Following ischemic stroke or intracerebral hemorrhage, the ECM undergoes a profound and dynamic remodeling, tightly linked to neuroinflammatory cascades and BBB disruption. Early after insult, immune cell infiltration and reactive gliosis lead to substantial ECM deposition, including collagens, proteoglycans (e.g. CSPGs and HSPGs), and tenascins, forming both fibrotic and astrocytic scars that demarcate injury zones while limiting plasticity in adjacent tissue (Dzyubenko et al., 2018; Chmelova et al., 2023). Activated endothelial cells and pericytes generate MMPs (MMP-2 and MMP-9) and proteases such as cathepsins, which degrade basement membrane proteins such as laminin IV and type IV collagen, contributing to BBB breakdown and formation of bioactive ECM fragments that further amplify inflammation. While initially detrimental, ECM degradation products and later upregulation of molecules such as thrombospondin-1 and biglycan may support regulatory T-cell responses, angiogenesis, and tissue remodeling during recovery phases. Thus, ECM modulation is a “double-edged sword” in stroke: it contributes to initial injury and inflammation but also holds potential to facilitate repair if precisely targeted (Li et al., 2023).

Among the different therapeutic strategies for stroke and intracerebral hemorrhage, the use of biomaterials and small-molecule modulators that aim to restore the ECM homeostasis has recently gained higher impact. Regarding the first approach, injectable ECM hydrogels derived from urinary bladder matrix were developed and implanted into stroke-induced brain cavities in rats. These ECM hydrogels were prepared at varying protein concentrations to evaluate the influence of their mechanical and biological characteristics on the therapeutic outcomes. Urinary bladder matrix hydrogels at 0, 3, 4, and 8 mg/mL were implanted into the lesion cavity 14 days post-injury. The lower concentration hydrogels (3 and 4 mg/mL) degraded significantly, losing about 95% of their volume by 90 days, while the more concentrated and mechanically stiffer 8 mg/mL hydrogel showed much slower resorption, with only 32% degradation over the same period. The less concentrated hydrogels allowed greater macrophage infiltration and accumulation within the scaffold, while macrophage presence was reduced in the denser hydrogel. Endothelial cell migration and new blood vessel formation were prominent in the 3 and 4 mg/mL hydrogels but absent in the stiffer analogues. All formulations supported progressive neural cell infiltration over time, with evidence of neural progenitor differentiation into mature neurons, displaying axonal projections, along with extensive oligodendrocyte presence. Astrocyte invasion was limited within the hydrogels but could be found in new tissue bridging degraded regions. Overall, the implantation of these ECM hydrogels encouraged partial neural tissue regeneration, though deeper insights are needed to fully assess their therapeutic potential (Ghuman et al., 2018).

Similarly, a recent study evaluated region-specific decellularized porcine brain extracellular matrix (dECM) as a biomaterial scaffold for *in vitro* models of ischemic injury, highlighting how ECM cues vary across brain subregions and impact neuronal behavior. The cortex-derived dECM supported robust neuronal attachment, survival, and maturation of PC12 and primary cortical cells, likely due to preserved neurotrophic factors (particularly brain-derived neurotrophic factor) and structural glycosaminoglycans critical for ECM signaling. These region-specific scaffolds demonstrated differential biochemical composition and neuronal outcomes compared to dECM from cerebellum or limbic regions, underscoring the importance of ECM location for effective remodeling. Tailoring dECM scaffolds to the specific brain region affected by stroke may allow for more precise ECM restoration, supporting selective neuronal populations and enhancing regenerative processes (Reginensi et al., 2020).

In ischemic stroke models, early inhibition of MMPs has been shown to preserve critical ECM and vascular structure, providing both neuroprotection and immunomodulatory benefits (Yang et al., 2015). For instance, administration of the broad-spectrum MMP inhibitor GM6001 shortly after middle cerebral artery occlusion in rats reduced degradation of basement membrane proteins (mainly laminin and type IV collagen), stabilizing tight junction components such as occludin and preserving BBB integrity. This early ECM preservation limited edema and cellular infiltration, while promoting neurovascular remodeling and increased angiogenesis during recovery (Yang et al., 2013). Similarly, SB3CT, a selective gelatinase (MMP-2 and MMP-9) inhibitor given after embolic stroke in mice, prevented the loss of laminin-positive pericytes (significantly increasing their number compared with vehicle controls containing PEG-200 and DMSO), alleviated ischemiainduced contraction of pericyte-surrounded capillary lumens, and sustained microvascular architecture in the peri-infarct region. These structural outcomes reduced infarct expansion, decreasing micro-hemorrhage and intracerebral hemorrhage volumes from more than 300 µm^3^ to less than 100 µm^3^ upon SB-3CT treatment, improving functional recovery. This suggests that maintaining ECM integrity via targeted MMP inhibition can modulate the stroke-induced neuroinflammatory cascade by preserving basal lamina networks and limiting secondary tissue damage (Cui et al., 2012).

Similarly, Katarzyna Greda and Nowicka (2021) demonstrated that targeting ECM remodeling through early inhibition of hyaluronidase activity can enhance post-stroke functional recovery in mice. Following ischemic injury, there was a rapid and simultaneous upregulation of both HA synthesizing and degrading enzymes in the perilesional cortex, indicating accelerated HA turnover and substantial ECM reorganization. Hyaluronidase 1 was predominantly localized to astrocytes, while HAS2 was found in both astrocytes and neurons, with post-stroke increases mainly in astrocytes. Additionally, β-glucuronidase shifted from neuronal to microglial expression after injury. Pharmacological inhibition of hyaluronidase with L-ascorbic acid 6-hexadecanoate, administered early after stroke, significantly improved skilled motor performance without altering PNN density. These findings indicate that following a stroke, there is significant restructuring of polysaccharide content, and disrupting this process during the early stages can positively influence recovery. Moreover, the selective modulation of HA metabolism, rather than the whole ECM degradation, can promote neural repair, highlighting hyaluronidase inhibition as a promising ECM-targeted therapeutic strategy for neurological disorders involving impaired plasticity.

As seen in TBI and SCI, enzymatic strategies can be used to restore the ECM and recover tissue homeostasis in stroke. In particular, ChABC has been applied to postischemic stroke to remove inhibitory CSPGs in PNN and glial scars, restoring neuroplasticity. In aged rat models of focal ischemic stroke, a delayed intraspinal injection of ChABC (three days post-event) significantly degraded CSPGs, induced contralesional corticospinal tract sprouting, and improved sensorimotor recovery, even when administered after the acute injury window (Soleman et al., 2012). Based on these findings, Hill et al. (2012) conducted a direct head-to-head comparison of three ECM-associated molecules: ChABC, glypican, and neurocan, infusing them in the infarct cavity of strokeinjured rats beginning seven days after injury. ChABC enzymatically cleaved inhibitory CSPGs and reduced GFAP-positive scar thickness (by approximately 40%), while restoring MAP2 immunoreactivity in the periinfarct cortex and improving motor recovery. Glypican, a heparan sulphate proteoglycan, provided a distinct mode of ECM modulation: without degrading CSPGs, it reduced astrogliosis, enhanced neurite outgrowth *in vitro*, and boosted expression of fibroblast growth factor-2, delivering permissive ECM cues that promoted structural plasticity and behavioral improvement. In contrast, neurocan infusion alone had no beneficial effects, reinforcing its status as an intrinsically inhibitory CSPG in the stroke ECM environment. These findings illustrate two complementary ECM-directed therapeutic strategies: (1) enzymatic removal of inhibitory matrix components via ChABC to lift physical and chemical growth constraints; and (2) supplementation with permissive molecules such as glypican to actively restore proregenerative ECM signaling.

The therapeutic use of ChABC is sometimes hindered by its low stability, which hampers its clinical application and translatability. To overcome this, recent innovations have emerged, including the delivery of more stable mutant forms of ChABC and/or their combination with hydrogels as drug delivery platforms. A relevant example of this is the mutant ChABC, ChASE37, that was encapsulated and released via an affinity-releasing carboxymethylcellulose hydrogel (Khait et al., 2025). The hydrogel matrix was formed via Diels–Alder click chemistry between carboxymethylcellulosenorbornene and carboxymethylcellulosetetrazine, producing a soft (approximately 1.1 kPa of elastic modulus), injectable, nonswelling network with tunable affinity sites that trap ChASE37 via reversible SH3–ligand binding. This architecture enabled the maintenance of ChASE37 activity over days (approximately 44% of activity retained at 7 days *in vitro*). Additionally, this material enabled prolonged focal CSPG digestion within stroke lesions, promoted axonal sprouting from human induced pluripotent stem cells-derived neurons *in vitro*, and remodeled the inhibitory ECM *in vivo* through sustained enzymatic release. Particularly, a significant increase in the digested CSPGs was observed in the ChASE37-treated (approximately 6% area of digested CSPGs) versus vehicle (approximately 0.5% area) and control (approximately 1% area) 7 days post injection. A similar approach was investigated by Hettiaratchi et al. (2019), through the engineering of a mutant ChABC variant (PEG-N1000G-ChABC-SH3), which was PEGylated to improve thermal stability and provide resistance to denaturation. These enzymes were delivered similarly using a methylcellulose hydrogel decorated with SH3 ligands, yielding an affinity-controlled release of enzymes over multiple days *in vivo*. As a result, a 49% CSPG reduction was observed in the perilesional penumbra at 14 days (approximately 40% at 28 days) poststroke in rats, along with a reorganization of glial scar ECM into a more permissive scaffold for plasticity and axonal reingrowth. These therapies show good promise for focal ECM-targeted therapy in ischemic stroke models and other neurological disorders involving neuroinflammation (Khait et al., 2025).

## Multiple Sclerosis

MS leads to significant remodeling of the ECM of the brain, shifting from a relatively loose, glycosaminoglycan-rich environment to a denser, more fibrotic structure. Moreover, in chronic MS plaques, aggregated Fn resists clearance, impairs oligodendrocyte precursor cells (OPC) maturation, and promotes a proinflammatory macrophage/microglial phenotype, further inhibiting lesion repair. Similarly, HMW HA accumulates within demyelinated lesions in MS, mainly by overproduction by HAS2, which is upregulated after an inflammatory insult in the cortex. This HMW-HA, largely deposited by reactive astrocytes, potently inhibits the differentiation of OPCs. It is believed that this is caused by the effect of hyaluronidases that fragment HMW-HA into smaller HA oligomers (200–400 kDa), which engage Toll-like receptor 2, activating inhibitory signaling that “locks” OPCs in an immature state and blocks remyelinating maturation (Sloane et al., 2010).

The effect of HA degradation using hyaluronidases or its synthesis inhibition on demyelination onset was deeply studied during the last decade. In an *in vitro* model, OPCs exhibited hyaluronidase activity expressing HYAL1, HYAL2, and PH20. Interestingly, only PH20 digestion products inhibited OPC maturation. On the contrary, OPC maturation was not affected by HA synthesis inhibition. Moreover, PH20 was overexpressed in OPCs and reactive astrocytes in a lysolecithin rodent model, and in human demyelinating lesions. Preston et al. (2013) claimed that the inhibition of PH20 may promote remyelination in the MS.

The inhibition of HA synthesis has been explored mainly by using 4-methylumbelliferone (4-MU) in MS-like models. 4-MU depletes glucuronic acid, then inhibits HA synthesis both *in vitro* and *in vivo* (Mueller et al., 2014). An oral dose of 4-MU was capable of ameliorating experimental autoimmune encephalomyelitis development in mice and rats, also suppressing the generation of encephalitogenic T-cell responses. Furthermore, the dose and pharmacokinetics of 4-MU were studied in depth by Kuipers et al. (2016a, b), which further proves the effect of 4-MU in inhibiting autoreactive T-cell activation in a murine experimental autoimmune encephalomyelitis model. A clinical trial aiming to study the optimal dose of 4-MU, also known as hymecromone, that would safely and effectively inhibit HA synthesis was assessed a few years back (NCT02780752). However, no results have been published to date.

**Figure 3:**
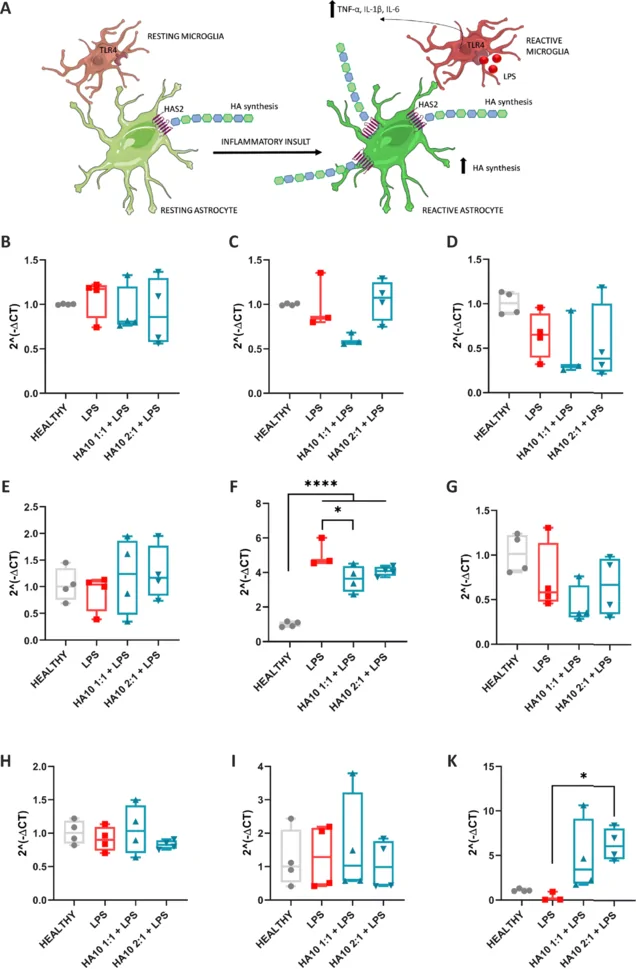
Source of externally cross-linked HA has a direct effect on key players in HA metabolism 7 days after treatment. (A) Schematic representation of the HAS2-mediated synthesis of HA enhanced after inflammation in the cortex. Neuroinflammation related to hyaluronan metabolism – gene expression fold change (B–D) HYAL 1, 2, and 3 respectively; (E–G) HAS1, HAS2, and HAS3 respectively; (H) CD44, (I) RHAMM, and (K) tumor necrosis factor-stimulated gene-6 (TSG6). Data are represented as mean ± standard deviation; *n* = 4; analysis of variance followed by Dunnet multiple comparisons test * < 0.05; ** < 0.01; *** < 0.005; **** < 0.001. Reprinted with permission from Martin-Saldaña et al. (2025) under a Creative Commons CC-BY License. CD44: Cluster of differentiation 44; HA: hyaluronic acid; HAS: hyaluronan synthase; HYAL: hyaluronidase; IL: interleukin; LPS: lipopolysaccharide; RHAMM: receptor for hyaluronic acid-mediated motility; TNF-α: tumor necrosis factor-alpha; TSG-6: tumor necrosis factor-stimulated gene 6.

A different approach was recently investigated by MartinSaldaña et al. (2025), by the development of crosslinked HA–based hydrogels (HA10, using a 4-arm PEG-NH_2_ crosslinker) and its effect on HA-related biomarkers in a complex *in vitro* cortical inflammation model of MS. In this study, it was found that these HA crosslinked scaffolds ameliorated intrinsic response to neuroinflammation by downregulating HAS2 (**Figure 3**). Particularly, the levels of HAS2 gene expression decreased, from a 5-fold increase after inflammatory insult to up to 3-fold increase after HA10 treatment, with respect to healthy controls (**Figure 3F**), demonstrating that introducing an external supply of crosslinked HA can reduce the unwanted HA synthesis by reactive astrocytes. Therefore, this could potentially prevent the excessive HMW-HA deposition commonly associated with ECM scarring on demyelinated axons. Additionally, these scaffolds reduced inflammatory signals (decreased tumor necrosis factor-α, IL-6, and other pro-inflammatory chemokines) along with an increase in IL-4 release, shifting glial cells toward anti-inflammatory phenotypes. Thus, the hydrogel not only provided the expected anti-inflammatory effect, but also actively recalibrated ECM synthesis and turnover, potentially mitigating inflammation-driven ECM remodeling. This mechanism aligns with a study using HA-based hydrogels in TBI/SCI models where modulation of HA metabolism has been seen to influence glial scar formation and inflammatory resolution (Martin-Saldaña et al., 2025).

Another important ECM component, which is tightly related to MS progression, is Fn. A comprehensive review by van Schaik et al. (2022) delves into fibronectin’s multifaceted roles in MS pathogenesis. Similar to HA, in MS lesions, Fn aggregation and accumulation contribute to an inhibitory lesion environment, hindering OPC differentiation and remyelination. Several therapeutic strategies to overcome Fn-mediated inhibition of remyelination in MS have been developed, aiming to control/modify the behavior and fate of Fn by different mechanisms, including: (1) preventing Fn expression, (2) preventing Fn aggregation, (3) degrading Fn aggregates, and (4) bypassing Fn aggregates. A summary of these therapeutic strategies and relevant studies is shown in **Table 3**.

**Table 3:** Therapeutic strategies to overcome the inhibition of remyelination failure caused by fibronectin aggregation

Strategy	Method	Mechanism of action	Reference
Prevent Fn expression	Inhibit tissue-transglutaminase 2	Reduce Fn expression and deposition	Espitia Pinzón et al., 2017; Espitia Pinzon et al., 2017
Prevent Fn aggregation	Modulate Fn splicing	Alter Fn conformation to favor cell-surface binding	Werkman et al., 2020
	Block TLR3 signaling in astrocytes	Prevent release of Fn fibrils from astrocyte surfaces	Werkman et al., 2020
	Modulate HSP90β activity	Disrupt unfolding of Fn dimers necessary for fibrillogenesis	Hunter et al., 2014
Degrade Fn aggregates	Increase MMP-7 expression/activity	Enzymatically cleave Fn and Fn aggregates	Wang et al., 2018
	Upregulate MMP-2/MMP-9 in astroglia infected with toxoplasma gondii	Astroglia expressing high levels of MMP-2 and MMP-9 degrade Fn in vitro, suggesting a clearance role beyond MMP-7	Lu and Lai, 2013
Bypass Fn aggregates	Ganglioside GD1a treatment	GD1a overcomes inhibition of OPC differentiation and enhances OPC proliferation, via activation of a PKA-dependent pathway	Qin et al., 2017
	Phosphodiesterase inhibitors	Prolong cAMP levels, activate PKA pathways that enhance CNS remyelination	Medina-Rodríguez et al., 2017

Data are sourced from van Schaik et al. (2022). cAMP: Cyclic adenosine monophosphate; CNS: central nervous system; Fn: fibronectin; HSP90β: heat shock protein 90 beta; MMP: matrix metalloproteases; OPC: oligodendrocyte precursor cells; PKA: protein kinase A; TLR3: Toll-like receptor 3.

To implement these mechanisms, different materials can be used, including hydrogels, small-molecule modulators, and enzymes. A relevant example of these strategies consists of the use of MMPs, such as MMP7, which could be used in MS lesions to degrade inhibitory Fn aggregates. Wang et al. (2018) demonstrated that MMP7 (notably secreted by M2-like microglia/macrophages) is downregulated in chronic lesions, which allows persistent Fn aggregates to block OPC maturation. In fact, MMP7 was able to cleave the inhibitory Fn aggregates into small approximately 13 kDa/ approximately 16 kDa fragments containing the alternatively-spliced “extra domain A” (EDA, also called EIIIA), a domain normally present in cellular Fn that mediates cell adhesion, signaling, and inflammatory responses. Therefore, upregulating MMP7 or activating its expression in microglia rescues this phenotype by cleaving Fn aggregates, effectively dismantling ECM barriers to remyelination. This aligns with previously discussed analyses in stroke, TBI, and SCI, where MMP-mediated ECM cleavage provides both barrier reduction and generation of bioactive matrix fragments to enhance tissue repair.

Overall, MS is characterized by maladaptive ECM remodeling, combining deposition of inhibitory matrix proteins, failure to clear scarring components, and persistent immune activation, which collectively block remyelination and foster chronic inflammation. Therefore, the field is shifting towards developing targeted therapies that not only address the inflammatory components of MS but also focus on modulating the ECM to promote remyelination.

## Alzheimer’s Disease

In AD, neurodegeneration is closely intertwined with dysregulated ECM dynamics as described in previous sections. In AD, post-mortem and animal model data reveal upregulated levels of ECM components such as collagen IV, Fn, perlecan, laminin, and specific proteoglycans, often converging at amyloid plaques and perivascular spaces, indicating a pathological remodeling of basement membranes and neural ECM during early disease stages. Meanwhile, PNNs remain surprisingly intact even within plaques, suggesting that some ECM components are preserved while others are disrupted, contributing to altered synaptic plasticity and impaired Aβ clearance pathways (Lepelletier et al., 2017).

**Figure 4:**
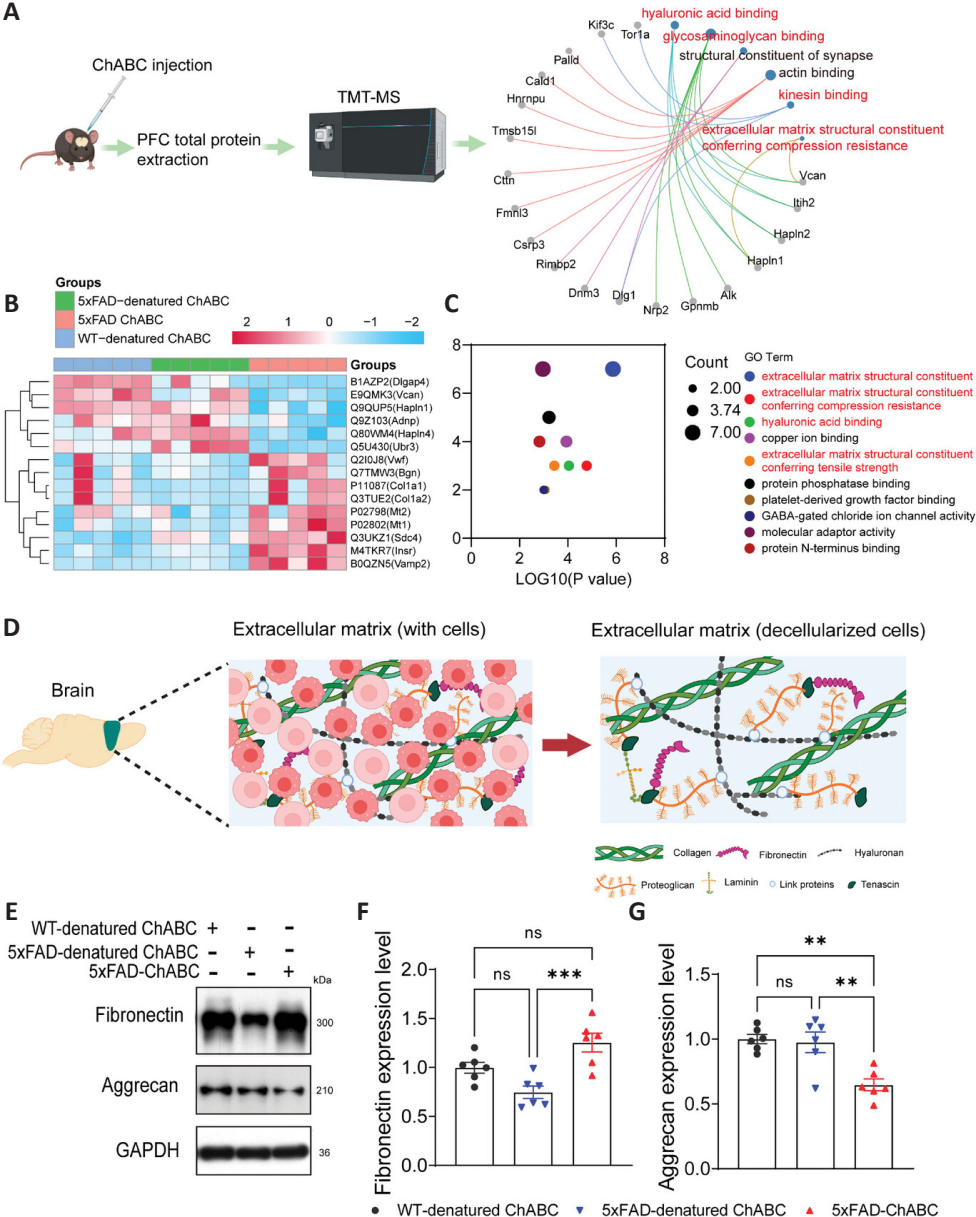
ChABC promotes extracellular matrix remodeling in the medial PFC of Alzheimer’s disease model mice. (A) Left: Experimental design schematic. Right: Significantly enriched GO terms identified from proteomic analysis of differentially expressed proteins in WT mice following treatment with active or denatured ChABC. (B) Heatmap displaying the top 15 proteins with differential expression identified via proteomics after ChABC or denatured ChABC treatment. (C) Bubble chart showing the top 10 significantly enriched GO terms related to differentially expressed proteins in 5×FAD mice following ChABC or denatured ChABC treatment. (D) Illustration of the brain cell decellularization procedure. (E) Western blot analysis of fibronectin and aggrecan levels in the PFC after decellularization in WT-denatured ChABC, 5×FAD-denatured ChABC, and 5×FAD-ChABC groups. (F, G) Quantification of fibronectin (F) and aggrecan (G) protein levels from the blots in the panel. Reprinted with permission from Yang et al. (2024) under a Creative Commons CC-BY License. ChABC: Chondroitinase ABC; GAPDH: glyceraldehyde 3-phosphate dehydrogenase; GO: Gene Ontology; ns: not significant; PFC: prefrontal cortex; TMT-MS: tandem mass tag-mass spectrometry; WT: wild type.

Regarding AD treatment approaches, a landmark study by Yang et al. (2015) demonstrated that intracortical injection of ChABC into the perirhinal cortex of tautopathy (AD) model mice degraded PNNs, resulting in full restoration of object recognition memory and synaptic transmission. Importantly, these effects occurred without altering the underlying neurodegenerative tau pathology, underscoring that targeted removal of inhibitory ECM components can restore neural plasticity and rescue cognition in AD models (Yang et al., 2015). In a more recent advance, Yang et al. (2015) used ChABC injections into the medial prefrontal cortex of 5×FAD mice (amyloid model), observing not only reversal of short-term memory deficits and reduction in Aβ deposition, but also ECM remodeling (including HA binding, GAG binding, kinesin binding, and ECM structural constituent conferring compression resistance), while decreasing aggrecan levels in plaques (**Figure 4**). Specifically, ChABC cleaves CSPG side chains on proteoglycans such as aggrecan, brevican, and neurocan, generating lectican fragments and reducing overall lectican burden in the periplaque matrix. Proteolytic trimming of CS downregulates CSPG-rich PNNs, reducing their compression resistance and loosening the ECM peri-plaque microenvironment. Simultaneously, no apparent upregulation of Fn, collagen IV, or laminin was detected, indicating a shift towards a looser ECM rather than the formation of a fibrotic tissue. Additionally, this treatment could induce astrocyte recruitment to amyloid regions, and enhance astrocytic autophagy‐lysosome activity, promoting plaque clearance. This study reveals that enzymatic ECM degradation can reprogram the peri-plaque microenvironment, facilitating both cellular phagocytosis and matrix-linked Aβ removal (Yang et al., 2024).

Complementing enzyme-based approaches, the emerging biomaterial strategy involves engineering biomimetic brain ECM scaffolds. Particularly decellularized dECM hydrogels derived from porcine brain, enriched with collagen, laminin, and remaining GAGs, showed that such biomimetic scaffolds significantly enhance neurite outgrowth and neural differentiation compared to collagen-only controls. Although direct Alzheimer applications are limited thus far, *in vitro* analyses demonstrate that injectable dECM hydrogels, especially when enriched with factors such as basic fibroblast growth factor, can support neurite outgrowth and shield neurons from Aβ toxicity, suggesting the potential of dECM to recreate a permissive ECM microenvironment in regions affected by AD pathology. These biomaterials preserve ECM components critical for neural development and might, in future *in vivo* models, reconstruct permissive ECM niches in AD lesions to support regeneration and mitigate Aβ toxicity (Yaldiz et al., 2021).

## Parkinson’s Disease

In PD, although the ECM has historically received less attention, recent omics-based investigations have highlighted widespread dysregulation of ECM-related gene networks. Transcriptomic, proteomic, and genomic analyses consistently report altered cell and focal adhesion pathways, integrin signaling, and glycoprotein expression in substantia nigra and affected regions, implying that ECM structural reorganization and cell-matrix interactions are changed in PD and may contribute to dopaminergic neuron vulnerability and neuroinflammation (Chapman and Sorg, 2024).

In PD models, recent analyses have leveraged ECM-mimetic biomaterials to enhance graft survival and integrate transplanted dopaminergic neurons. Penna et al. (2022) used self-assembling peptide hydrogels functionalized with a laminin epitope and stromal-derived factor 1, which mimic laminin-integrin signaling critical for cell adhesion and differentiation. Rats receiving fetal dopaminergic grafts within this scaffold had significantly larger graft volumes, increased A9-subtype dopamine neuron differentiation (3-fold increase in dopaminergic neurons after treatment), and enhanced reinnervation, indicating that ECM-like bioactive scaffolds can reshape the transplanted niche to support functional reconstruction (Penna et al., 2022). Similarly, Comini et al. (2024) demonstrated that a type I collagen hydrogel enriched with neurotrophic factors (glial-derived neurotrophic factor and brain-derived neurotrophic factor) dramatically improved the outcome of human induced pluripotent stem cells-derived dopaminergic progenitor transplantation. Compared to suspension controls, the hydrogel group had an approximately 8-fold increase in cell survival, an approximately 16-fold increase in tyrosine hydroxylase–positive neuron differentiation, and reduced gliosis at the graft site. These data confirm that augmenting ECM-like structures with adhesive and trophic cues can fundamentally alter the graft microenvironment, facilitating dopaminergic maturation and integration in PD brains (Comini et al., 2024).

On a different approach, Zhang et al. (2025b) used small-molecule inhibitors of colony-stimulating factor 1 receptor, a tyrosine kinase expressed primarily on microglia and macrophages whose activation regulates their survival, proliferation, differentiation, and homeostasis in the brain. In this study, the inhibitor PLX5622 was administered orally 3 weeks prior injection of rAAVhSYN, an adeno‐associated virus vector used to induce Parkinson’s-like disease by promoting the overexpression of α-synuclein selectively in nigral dopaminergic neurons. The pre-treatment with PLX5622 prevented the development of motor deficits while preserving dopaminergic neuron cell activity. Additionally, the treatment affected the expression of multiple ECM-related genes and proteins (e.g., ctfg that encodes CCN2 protein, and nov that encodes CCN3 protein) in the affected brain regions, suggesting structural remodeling of the ECM toward a more regenerative state (Zhang et al., 2025b). Essentially, removing chronically activated microglia reduced inflammatory pressure on the matrix and promoted expression of ECM-regulatory pathways involved in tissue maintenance and repair.

When compared with biomaterial and small-molecule-based methods in PD and AD, it becomes clear that both immune-cell modulation and ECM-inspired biomaterials share a common goal: to reprogram a hostile ECM environment into one that supports neuronal survival, integration, and function.

Across models of TBI, SCI, and experimental autoimmune encephalomyelitis, a novel therapeutic target is increasing attention, focusing on the role of the ECM not only in tissular structural integrity but also in modulating inflammation, regeneration, and functional outcomes. Enzymatic approaches such as ChABC effectively degrade inhibitory CSPGs, mitigating glial scarring and enhancing axonal regeneration and synaptic plasticity. Simultaneously, HA-targeted strategies, whether through degradation with hyaluronidase to reduce edema or incorporation of HA-based hydrogels for immunomodulation, show how both the physical and biochemical properties of the ECM influence recovery. Biomaterials such as HA methacryloyl hydrogels, delivering IL-10 or hydrogels engineered to incorporate exosomes and ECM cues, further illustrate the potential of scaffold-based therapies to reshape the lesion environment. Collectively, these studies underscore the therapeutic promise of ECM-modulating strategies that act not only as structural supports but as active regulators of the neuroinflammatory and regenerative landscape following CNS trauma.

## Future Perspectives

In the 19^th^ century, Santiago Ramon y Cajal provided the foundation of modern neuroscience by using Camillo Golgi’s stain with a detailed description of nerve cell organization in the brain (Ramón y Cajal and Azoulay, 1955). Despite their disagreement in CNS organization, Golgi and Ramon y Cajal established the principles of the basic understanding of CNS and PNS milieu. However, the ECM-focused analyses were underseen for more than a century, until the decade of the 1960s, when the presence of periodic acid–Schiff positive material around nerve cells was observed in vertebrate brains (Celio et al., 1998). This periodic acid–Schiff positive material was nothing but the negatively charged components of the PNNs, namely CSPGs, glycoproteins, and HA (Celio et al., 1998).

In the last 30 years, the body of research about ECM in the CNS has blossomed, and our knowledge on the critical role of its components in shaping the biological milieu of the CNS, as well as in a neurodegenerative context, is continuously evolving. However, we still do not fully understand if these changes are a cause or a consequence of the CNS diseases.

**Figure 5:**
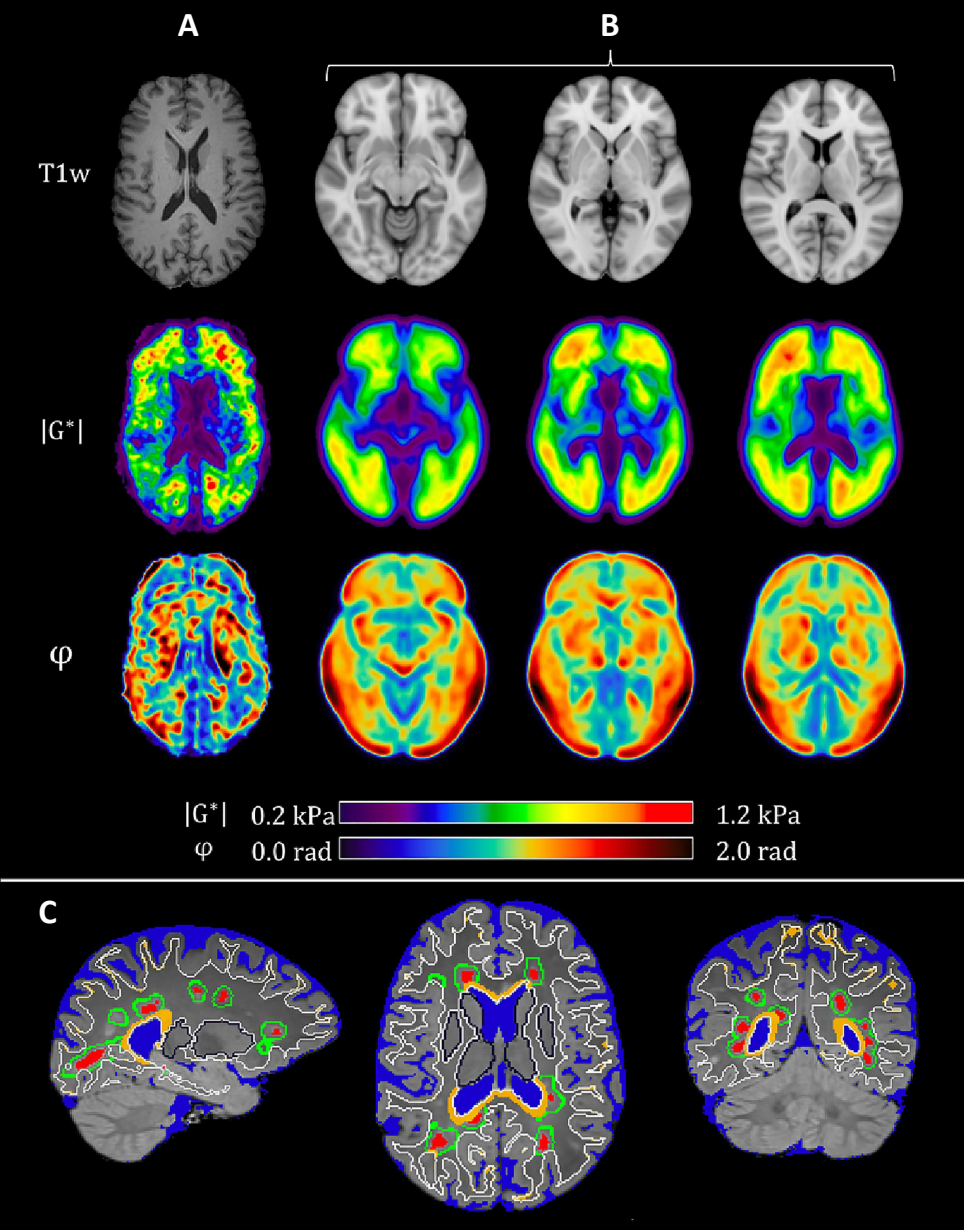
Low-frequency magnetic resonance elastography revealed altered deep gray matter viscoelasticity in multiple sclerosis. (A, B) Viscoelasticity maps visualization; (C) lesion/rim mask creation. (A) Representative maps of G* and φ of an multiple sclerosis patient with corresponding T1 weighted (T1w) image for anatomical reference. (B) Three axial maps of and representing voxel-wise means of all patients after transformation to MNI152 space with corresponding T1w images for anatomical reference. Z from left to right (world coordinates): –5, 0, 5. (C) Sagittal paramedian, axial, and coronal views (left to right) of manually delineated lesion masks (red) and their calculated corresponding rim masks (green) in a multiple sclerosis patient. Only voxels both overlapping with normal-appearing white matter (white outline) and at a distance greater than 3 mm to cerebrospinal fluid (dark blue, distance depicted in orange) were included. The basal ganglia and thalamus are delineated with a black outline. Each lesion and its corresponding rim represent an individual mask, enabling assignment to a white matter lobe. Reprinted with permission from Kiss et al. (2024).

As we have discussed in this review, the potential of ECM as a diagnostic biomarker, and even as a fingerprint of disease prognosis, is a reality. The changes in composition, degradation, or overproduction are emerging as diagnostic markers for various neurological conditions, including Alzheimer’s disease, Parkinson’s disease, and MS. However, this potential is slowed down by the need for advanced techniques that would allow clinicians to efficiently image the ECM *in vivo* since the knowledge about ECM changes comes mostly from post-mortem analysis (Ge et al., 2024; Di et al., 2025). Advances in magnetic resonance imaging (Digilio et al., 2023), positron emission tomography (Beziere et al., 2019; Isser et al., 2023), or the development of new imaging probes (Fiore et al., 2025) would allow clinicians to study ECM changes in the inflammatory disorders, further exploiting its power as a diagnostic marker. In this sense, a new living imaging method was recently developed and tested in preclinical models of glioma and stroke (Ge et al., 2024). The method, named pan-ECM, chemically labels ECM proteins and efficiently binds all the major components in BM, PNNs, and interstitial ECM. Furthermore, authors assessed pan-ECM efficacy in imaging *in vivo* the ECM heterogeneity between the glioma core and margin, and ECM, dismantling in stroke. Altogether, pan-ECM exhibited a promising potential for ECM *in vivo* imaging with optical resolution. Moreover, some techniques, such as magnetic resonance elastography, also exhibited potential to further study sex differences in CNS disorders that would allow for designing patient-specific therapies (Arani et al., 2021; Kiss et al., 2024). Kiss et al. (2024) recently found age-independent alterations in MS patients at early stages of disease compared with healthy volunteers when analyzed their brains by magnetic resonance elastography (**Figure 5**). Moreover, their results exhibited effects associated with disability that were not dependent on the duration of the disease. Some of the results achieved challenge previous reports on tissue remodeling in MS and their reflection in viscoelastic parameters, opening the discussion for further analysis and comparison among different techniques (Kiss et al., 2024).

Regardless of being the cause or the consequence of neurodegeneration, several pre-clinical analyses discussed here have shown the potential of ECM components as therapeutic targets aiming to tackle CNS disorders. Despite these advances, the clinical translation of ECM-targeted therapies is still flourishing. Most of the current clinical trials focused on ECM remodeling in the CNS are focused on disorders involving BBB, or bloodspinal cord barrier disruption, according to clinicaltrials.gov. The ongoing phase 2 clinical trial, “MATRISS-II” (NCT06700824), is evaluating the safety and efficacy of OTR4132 in patients who have experienced an acute ischemic stroke; to date, 60 patients have been enrolled. OTR4132 is a device made of synthetic polysaccharides with a dextran backbone designed to mimic HS with improved resistance to enzyme degradation. In preclinical models of stroke, OTR4132 exhibited neuroprotective effects and reduced the lesion volume (Pereira et al., 2022). It was also shown that a fluorescein-labelled OTR4132, injected via the intracerebroventricular or intramuscular route immediately after injury, accumulated specifically at the lesion site. It also reduced the lesion volume and promoted functional recovery in a rat model of transient ischemic stroke (Khelif et al., 2018). Other therapeutics out of the scope of this article, which are currently in different phases of clinical trials aiming to recover BBB or bloodspinal cord barrier integrity, have been thoroughly revised by Lemarchant et al. (2025).

In MS, the clinical trial entitled “TREAT-MS” (NCT03500328) aims to evaluate the effect of an early aggressive therapeutic approach involving hyaluronidase versus the traditional first-line therapy. The trial assesses the comparison between the two approaches on the relapsing remitting phase of the disease, hypothesizing a reduction in the intermediate-term disability in patients receiving an early aggressive therapy. Thus, patients would receive any of the traditional treatments (fingolimod, onazimod, dimethyl fumarate, and glatiramer acetate) or an aggressive approach, including ocrelizumab and hyaluronidase-ocsq (Ocrevus Zunovo), with doses of 920 mg ocrelizumab and 23,000 U hyaluronidase subcutaneously administered for 6 months.

Additionally, HyQvia is a formulation based on hyaluronidase-facilitated subcutaneous 10% immunoglobulin administration that has been proven to be safe in infants, adults, and elderly people (Paassen et al., 2020; Wasserman and Group, 2020). In this sense, a recently completed phase 3 clinical trial (NCT02549170) and its open-label long-term extension (NCT02955355) studied the effect of HyQvia in chronic inflammatory demyelinating polyradiculoneuropathy. In brief, 138 participants received either HYQVIA/HyQvia or the 0.25% albumin placebo solution with recombinant Human Hyaluronidase (rHuPH20) for 6 months or until relapse. The primary outcomes consisted of evaluating safety, tolerability, and immunogenicity, while efficacy was an exploratory outcome. Patients received doses every 3 or 4 weeks, and overall, a favorable long‐term safety and tolerability, as well as a low relapse rate, were demonstrated, which supports use as maintenance CIDP treatment. In this particular context, rHuPH20 is used to increase the permeability of the IgG, but it supposes a promising safety profile to be used as the key treatment in other diseases involving demyelination (Hadden et al., 2024). Moreover, there are 295 clinical trials at different stages involving the use of hyaluronidase for a wide variety of conditions, further proving its therapeutic potential. Other promising therapeutics, such as ChABC or 4-MU, are still accumulating pre-clinical evidence that would potentially put them in the pipeline for clinical trials in the upcoming years.

However, ECM therapies currently present some limitations, mostly due to the inherent complexity of the CNS ECM and the limited knowledge we still have. We still do not fully understand the long-term effect, as well as the potential undesired side effects, of using ECM-modifying therapies such as 4-MU, hyaluronidases, or ChABC. Furthermore, most of the murine models of the main neurodegenerative disorders present limitations (Zhang et al., 2025a). Therefore, the translation of these potentially disrupting treatments to clinics remains challenging. Longer *in vivo* analyses are needed, both in murine and large animal models, assessing the long-term outcome of these treatments, aiming to study the ECM remodeling phenomena.

The knowledge about ECM has become deeper in the last years thanks to glycobiology analyses. The changes in glycosylation, or post-translational addition of glycans to protein backbones, in healthy CNS and after neuroinflammatory-driven disorders are gaining increased interest. Understanding the glycan fingerprint of CNS disorders would allow us to identify targets potentially addressed by newly developed glyco-based therapeutics (Rebelo et al., 2021). Additionally, studying the changes that cells are experiencing during the ECM remodeling in disease, as well as after the treatment, would provide invaluable information to predict the fate of the overall CNS homeostasis recovery.

Nevertheless, to improve the chances of translation towards the clinical scenario, clinicians, neuroscientists, and chemists need to work closely to understand the intricate processes that occur during ECM turnover and remodeling that can be therapeutically modulated. While the complexity of CNS disorders means that targeting a single ECM component is unlikely to provide a standalone cure, combining such approaches with existing or emerging therapies offers great promise for reshaping the CNS environment in ways that support recovery. Encouragingly, our knowledge of both the chemistry and biology of ECM in healthy CNS and in neurodegeneration continues to expand, paving the way for innovative strategies and a promising future.

## Data Availability

*Not applicable.*
